# Prevention and Management of Spinal Cord Ischemia After Aortic Surgery: An Umbrella Review

**DOI:** 10.3390/brainsci15040409

**Published:** 2025-04-17

**Authors:** Alexandros G. Brotis, Adamantios Kalogeras, Metaxia Bareka, Eleni Arnaoutoglou, Kostas Spanos, Miltiadis Matsagkas, Kostas N. Fountas

**Affiliations:** 1 Department of Neurosurgery and Medical, Faculty of Medicine, School of Thessaly Health Sciences, University of Thessaly, 41500 Larissa, Greece; 2 Department of Neurosurgery, University Hospital of Larissa, 41500 Larissa, Greece; kalogadam@gmail.com; 3 Department of Anaesthesiology, Medical Faculty of Medicine, School of Thessaly Health Sciences, University of Thessaly, 41500 Larissa, Greece; barekametaxia@hotmail.com (M.B.); earnaout@gmail.com (E.A.); 4 Department of Vascular Surgery, Medical Faculty of Medicine, School of Thessaly Health Sciences, University of Thessaly, 41500 Larissa, Greece; spanos.kon@gmail.com (K.S.) mimats@uth.gr (M.M.); 5 Department of Neurosurgery, Medical Faculty of Medicine, School of Thessaly Health Sciences, University of Thessaly, 41500 Larissa, Greece; fountas@uth.gr

**Keywords:** spinal cord ischemia, aortic surgery, incidence, risk factors, prevention, early detection, treatment, prognosis, umbrella review

## Abstract

**Background/Objectives:** Spinal cord injury is a devastating complication of aortic surgery, with significant morbidity and mortality. This review aimed to summarize the current literature on preventing and managing spinal cord ischemia after open and endovascular aortic repair. **Methods**: We conducted a comprehensive review of PubMed, Scopus, and the Web of Science, focusing on systematic reviews and meta-analyses of the pathophysiology, risk factors, and strategies for mitigating the risk of spinal cord injury after aortic repair. We assessed the quality of the reporting for the eligible studies using the AMSTAR-2 tool and evaluated the strength of the evidence using the GRADE approach. Due to the absence of homogeneous clinical data, the evidence was synthesized in a narrative form. **Results:** Spinal cord ischemia can occur after both open and endovascular aortic repair, with a higher incidence reported in more extensive thoraco-abdominal aortic aneurysm repairs. The underlying pathogenesis is largely understudied. Several preventive strategies have been partially investigated, including cerebrospinal fluid drainage, hypothermia, and distal aortic perfusion. While the employment of neuromonitoring has been established in spine surgery, its efficacy in aortic repair remains uncertain due to confounding factors like hypothermia, anesthesia medications, and cardiopulmonary bypass. The prompt management of spinal cord complications is crucial to optimizing outcomes. No clear treatment algorithm has been universally adopted. **Conclusions:** Spinal cord ischemia remains a major challenge in aortic surgery, with a significant impact on patient outcomes. Further research is needed to elucidate the relevant pathophysiology and develop more effective intraoperative monitoring and management strategies.

## 1. Introduction

Spinal cord ischemia (SCI) is a devastating complication, which can occur after aortic surgery, resulting in permanent paralysis and significant patient morbidity [1,2,3]. Multiple factors, including the extent of aortic disease, the duration of circulatory arrest, and the techniques of cerebral and spinal cord protection, have been implicated in the development of this complication [4,5,6].

Although the literature on the management of SCI, including optimal perfusion and temperature strategies, is extensive, it remains a subject of ongoing debate [4,5,6]. Significant variations exist regarding the actual complication’s incidence and predisposing risk factors across published studies. The existing knowledge on the early prediction of ischemia is also limited. There is an ongoing debate regarding the benefit of preventive measures such as cerebrospinal fluid drainage (CSFD), along with their potential complications [7,8]. Presently, there is no widely agreed-upon treatment for SCI [9].

To address this gap, we performed an umbrella review of the available evidence to provide an overview of the current state of knowledge and identify areas for future research. This study aims to provide a comprehensive overview of the current knowledge regarding SCI following aortic surgery based on systematic reviews and meta-analyses. The primary objectives are to summarize the incidence risk factors (Q1), pathogenesis (Q2), methods for early diagnosis (Q3), and management strategies, including preventive measures (Q4) and the potential complications associated with CSFD (Q5), as well as the treatment (Q6) and prognosis (Q7) of this devastating complication (Table 1). We will also discuss potential sources of heterogeneity across studies and make recommendations for future research directions.

This review will be of interest to vascular and cardiothoracic surgeons, neurologists, and neurosurgeons, as well as anesthesiologists and critical care physicians involved in managing patients undergoing aortic surgery.

## 2. Materials and Methods

### 2.1. Study Design

This umbrella review provides a comprehensive overview of available systematic reviews and meta-analyses on SCI following aortic surgery. The study was conducted and reported in alignment with the Preferred Reporting Items for Systematic Reviews and Meta-Analyses (PRISMA) guidelines. This secondary research synthesis did not require ethical approval, as it did not involve direct patient involvement. Additionally, this project was conducted without external funding.

### 2.2. Information Sources

From inception to December 2024, two review authors (A.B. and A.K.) systematically searched several electronic databases, including PubMed, Scopus, and Web of Science, for relevant systematic reviews and meta-analyses. We also searched the reference lists of included studies for potential additional references. Additionally, the authors searched for gray literature, such as conference proceedings and unpublished studies, to minimize the risk of publication bias.

### 2.3. Search Strategy

The search strategy combined keywords related to SCI, aortic surgery, and the study design (systematic reviews and meta-analyses). The full search strategy was (“spinal cord ischemia” OR “neurological deficits” OR “spinal cord injury” OR paraplegia OR paraparesis) AND (“endovascular aortic repair” OR “abdominal aortic surgery” OR “aortic aneurysm”) AND “(management” OR “treatment” OR “hypothermia” OR “cerebrospinal fluid drainage” OR “intercostal artery reimplantation”), with modifications according to the database requirements.

### 2.4. Study Selection

**Figure 1:**
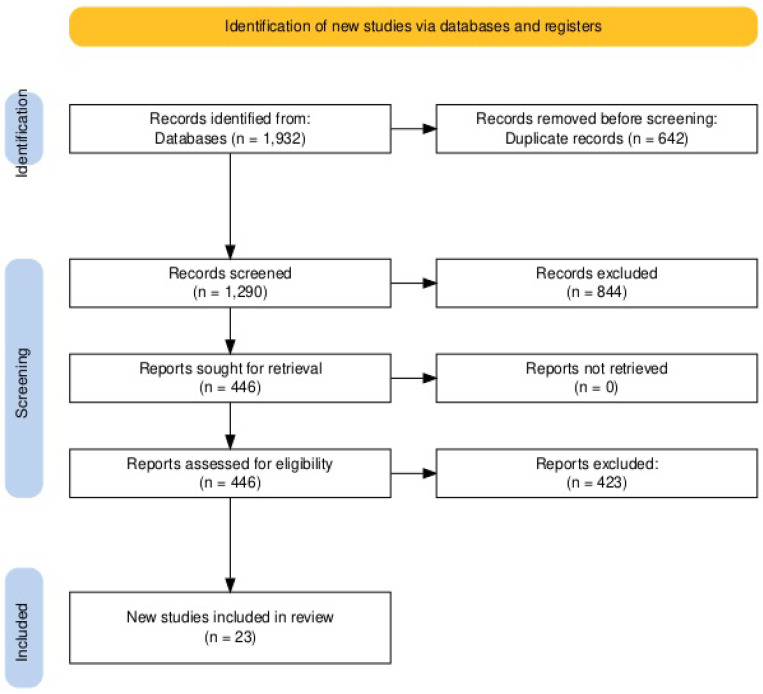
Prisma flow chart of our literature search.

Two review authors (A.B. and A.K.) independently screened the titles and abstracts of identified studies, retrieving full-text articles for further assessment. Studies were included if they met the following criteria: (1) systematic review or meta-analysis; (2) focused on SCI following aortic surgery; (3) reported at least one of the following parameters: incidence, risk factors, early diagnosis, prevention, treatment, or prognosis; and (4) written in English. Narrative reviews, expert opinions in languages other than English, and individual primary studies were excluded. The authors used the Rayyan web application for collaborative reviews during the screening process [10]. In case of disagreement, the authors consulted a senior author (K.N.F.) (Figure 1).

### 2.5. Data Extraction

Two review authors (A.B. and A.K.) independently extracted relevant data using a pre-designed data extraction form from the included studies. The extracted data included study characteristics (authors, year of publication, study design, number of included studies, and total number of patients), details on the population, intervention or exposure, comparison, and outcomes. We additionally extracted the author’s search keywords, databases, eligibility criteria, and quality assessment methods.

### 2.6. Data Synthesis

For qualitative data, the evidence synthesis for this umbrella review will be presented in a narrative format, providing a comprehensive overview of the key findings from the included systematic reviews and meta-analyses. The narrative synthesis will summarize the incidence, risk factors, diagnostic methods, management strategies, and prognosis of SCI following aortic surgery. Relevant data, such as pooled estimates, will be presented in table format to aid in the interpretation of the results. The narrative review will also discuss potential sources of heterogeneity across the included studies and will highlight areas for future research to address the existing knowledge gaps in this field. For quantitative data, where appropriate, we conducted umbrella meta-analyses using the “metaumbrella” package for R version 4.3.3 to provide pooled estimates of the incidence of SCI, the effectiveness of specific management strategies, and the risk associated with various risk factors. The “metaumbrella” package allows for the synthesis of multiple meta-analyses, enabling us to provide a comprehensive overview of the quantitative evidence on this topic. This approach will help identify patterns and sources of heterogeneity across the included studies.

### 2.7. Quality Appraisal

Two review authors (A.B. and A.K.) appraised the gathered literature using AMSTAR-2 [11,12]. The quality appraisal of the literature using AMSTAR-2 involved a rigorous assessment of systematic reviews based on 16 key domains. These domains cover crucial aspects of review methodology, including protocol registration, search strategy comprehensiveness, risk of bias assessment, and methods of data extraction [11,12]. Seven of these domains were designated as “critical”, carrying greater weight in the overall appraisal. AMSTAR 2 does not generate a numerical score, but provides an overall rating of “high”, “moderate”, “low”, or “critically low” based on the number of “no” responses for each domain. This rating reflects the methodological rigor and trustworthiness of the reviewed systematic reviews, aiding in the identification of high-quality evidence for clinical decision-making and research [11,12]. The results were visualized using amstar2Vis [11,12].

The overall quality of the output evidence was assessed according to GRADE recommendations [13]. The GRADE system is a widely used framework for evaluating the certainty of evidence in systematic reviews and meta-analyses [13]. It assesses the certainty of evidence on a scale from high to very low, based on factors such as risk of bias, inconsistency, indirectness, imprecision, and publication bias [13].

## 3. Results

### 3.1. Literature Search

The systematic literature search yielded a total of 1932 records. After removing duplicates, 1290 unique citations were screened. Of these, 121 full-text articles were assessed for eligibility, and 23 systematic reviews and meta-analyses were included in the final synthesis.

### 3.2. Study Characteristics

The included studies were published between 2004 and 2024 (Table 2). Sixteen employed quantitative meta-analysis, while six were qualitative in nature. The most frequently utilized databases were PubMed Ovid, Cochrane Library, ClinicalTrials.gov, Embase, Scopus, CINAHL, and Embase. The study population comprised patients undergoing open aortic surgery with EVAR and TEVAR for thoraco-abdominal aortic aneurysms (TAAA) or dissections. The most common prophylactic measure was CSFD.

### 3.3. Incidence and Risk Factors (Q1)

Twelve studies have examined the incidence of SCI as a primary or secondary focus (Table 3), six of which comprised the primary question. However, the included studies exhibited significant heterogeneity in their techniques, case selection, and outcome measures, leading to a wide range of reported incidences, from as low as 0% to as high as 33%.

The overall incidence of SCI after surgical and endovascular aortic repair is about 10% and 3.5%, respectively [3,4,7,9,14,19,20,21] (Table 4). However, permanent deficit remains in approximately 3.3%, while late events are rare and limited to less than 2% of the cases [3,4,7,9,14,19,20,21,23,25].

A systematic review and meta-analysis by Rocha et al. found a higher overall incidence of SCI following endovascular TAAA repair (13.5%) compared to open repair (7.4%) [4]. Yet, the occurrence of permanent spinal cord injury was comparable, with a combined incidence of 5.2% for endovascular and 4.4% for open repair [4].

According to a systematic review by Lella et al. (2022), the incidence of SCI varies depending on the type of aortic repair. For endovascular descending thoracic aortic repair, the overall SCI rates ranged from 0% to 10.6%, with permanent SCI ranging from 0% to 5.1% [9]. For endovascular thoraco-abdominal aortic repair, the overall SCI rates ranged from 0% to 35%, with permanent SCI ranging from 2% to 20.5% [9]. In the case of open thoraco-abdominal aortic repair, one study reported a permanent SCI rate of 1.1%, while the overall SCI rates were described as being within the 0–35% range, although not explicitly stated [9].

The study by Pini et al. reported an overall pooled incidence of SCI of 11% after endovascular TAAA repair [20]. However, the incidence varied based on the extent of the TAAA repair, with extent IV repairs having a pooled rate of 6%, while repairs involving extents I–III and V had a higher rate of 13% [20]. Although there was a trend towards lower SCI rates in staged procedures, the difference was not statistically significant [20]. Additionally, the study explored other potential risk factors, such as age and aneurysm diameter, but did not find consistent associations with SCI [20].

Muston et al. examined SCI as a primary outcome in staged TAAA repairs, comparing open, endovascular, and hybrid approaches [3]. They found an overall pooled SCI incidence of 5.4% across all staged repairs [3]. While there was a trend towards lower SCI rates with the hybrid approach (3.2%) compared to open (1.4%) and endovascular (9.8%), these differences were not statistically significant [3]. The study highlighted the challenges in comparing SCI rates due to variations in reporting across studies and emphasized the need for standardized reporting in future research [3].

According to a meta-analysis by Alzghari et al., the pooled incidence of permanent spinal cord injury was 3.3% [2]. This incidence varied based on the surgical approach, with 4% for open repairs and 2.9% for endovascular repairs [2]. Additionally, the permanent SCI rate differed by the location of the aneurysm, being 2.0% for descending thoracic aneurysm repair and 4.7% for TAAA repair [2]. The permanent SCI rate also varied with the extent of the TAAA, ranging from 3.8% for Crawford extent I to 13.4% for extent II, 7.1% for extent III, 2.3% for extent IV, and 6.7% for extent V [2].

Separately, a meta-analysis by Zheng et al. in 2024 reported a pooled estimated risk for permanent SCI after TEVAR of 2.0%, based on 22 studies with 1479 patients [6]. The pooled estimated risk for temporary SCI was 1.0% based on 24 studies with 2048 patients [6].

The incidence of delayed SCI after aortic surgery was examined indirectly by two eligible studies, only as a secondary question. In a systematic review by Sef et al., the reported incidence of delayed SCI varied considerably, ranging from as low as 1% to as high as 12% across the included studies [23]. This variation likely reflects differences in patient populations, surgical techniques, and definitions of delayed SCI used across the included studies [23]. This systematic review underscored the challenges in determining a precise incidence due to these inconsistencies [23]. In a meta-analysis, Chen et al. investigated the relationship between prophylactic CSFD and SCI during thoracic and thoraco-abdominal endovascular aortic repair [21]. They reported incidences of 1.3% for immediate SCI, defined as the presence of paraplegia at the emergence of anesthesia, and 1.9% for delayed SCI, although the definition of delayed SCI was not clearly specified [21].

### 3.4. Pathogenesis (Q2)

Only the systematic review by Sef et al. discussed in depth the potential mechanisms underlying SCI after aortic surgery [23]. The proposed mechanism of neurologic injury following TA repair involves the disruption of the spinal cord’s blood supply at some point, leading to hypoxia [23]. Spinal cord edema and microthrombi development can further reduce perfusion pressure [23]. Existing evidence indicates that the spinal cord relies extensively on a collateral network, often as much as on any nominal vessel, allowing for sufficient blood flow even in the face of ischemia [23]. In addition, experimental data show that spinal cord perfusion pressure drops after segmental artery sacrifice, which gradually recovers over time, however [23].

### 3.5. Early Diagnosis (Q3)

Five studies examined the role of various diagnostic modalities in the early detection of SCI. The systematic review by Sef et al. (2023) explored the use of perioperative neuromonitoring during open TAAA repair [23]. The authors analyzed studies investigating different neuromonitoring methods, such as motor-evoked potentials (MEPs), somatosensory evoked potentials (SSEPs), and near-infrared spectroscopy (NIRS), to assess their ability to predict and prevent SCI [23]. MEPs emerged as the most commonly used and studied modality, demonstrating reasonable sensitivity and specificity for detecting SCI [23]. While SSEPs and NIRS were also investigated, the evidence supporting their use was less robust, with limitations in sensitivity and specificity [23]. The review suggested that a multimodal approach, combining different neuromonitoring techniques, might offer the best chance of detecting SCI [23].

Tanaka et al. conducted a systematic review and meta-analysis to establish the effectiveness of MEPs in predicting SCI during open surgical repair of thoracic and TAAAs [15]. MEPs had an estimated 77% pooled sensitivity in identifying cases of SCI, and a 95% pooled specificity in accurately ruling out the condition [23]. The diagnostic odds ratio was 30, indicating a strong association between MEPs changes and SCI. However, the authors noted substantial heterogeneity among the included studies, while the study’s methodological quality was moderate [23]. The authors concluded that MEPs constitute a valuable tool for predicting SCI during open TAAA repair, but its sensitivity may be limited [23].

The systematic review by Harky et al. investigated the potential of CSF biomarkers in predicting SCI after TAAA repair, including S-100β, neuron-specific enolase, lactate, glial fibrillary acidic protein A, Tau, heat shock proteins 70 and 27 (HSP70, HSP27), and pro-inflammatory cytokines [16]. The review found that, while several biomarkers showed potential, there is a lack of high-quality studies with consistent findings, suggesting limited evidence. Furthermore, commonly measured markers like lactate, S-100β, NSE, and Tau did not reliably correlate with SCI occurrence, indicating an inconsistent correlation with SCI [16]. However, their review identified GFAP, HSP70, HSP27, and IL-8 as promising parameters, as they showed significant increases in SCI patients, warranting further investigation [16].

Thet et al.’s review documented the current evidence on neuromonitoring during endovascular repair of descending thoracic aortic and TAAAs [25]. The review found that somatosensory-evoked potentials and motor-evoked potentials are the most frequently employed neuromonitoring techniques, offering reasonable sensitivity for identifying critical spinal cord injury [25]. The review also discussed other methods, such as near-infrared spectroscopy, but the evidence supporting their use is less established [25]. The review highlighted that neuromonitoring is particularly valuable in high-risk patients, enabling timely intervention to prevent or mitigate SCI [25].

Soliman et al. covered both established methods like motor-evoked potentials (MEPs) and somatosensory-evoked potentials (SSEPs), as well as newer modalities such as near-infrared spectroscopy (NIRS), computed tomography (CT), magnetic resonance imaging (MRI), and CSF biomarkers [26]. Although the perioperative use of MEPs and SSEPs is limited due to the lack of an established protocol, NIRS appears to offer the continuous, non-invasive monitoring of spinal cord oxygenation [26]. However, the effectiveness of NIRS is constrained by its shallow penetration depth and susceptibility to artifacts [26]. Similarly, CT and MRI offer detailed anatomy, but still, they are expensive, time-consuming, and lack real-time continuous recording [26]. Diffusion-weighted MRI shows promise for early SCI detection but cannot be used intraoperatively [26]. CSF and serum biomarkers have uncertain clinical utility [26]. The authors concluded that a multimodal approach combining various neuromonitoring modalities could be the most effective for postoperative SCI monitoring [26].

### 3.6. Prevention (Q4)

The review by Cina et al. showed that CSFD significantly reduces the risk of paraplegia after open aortic repair, with an absolute risk reduction of 10 [8]. With the use of prophylactic CSFD, one case of paraplegia is prevented for every 10 patients treated [8]. The review suggests that maintaining CSF pressure below 10 mmHg may be crucial for the effectiveness of CSFD in preventing paraplegia [8]. In conclusion, the review affirms that CSFD is an effective method for preventing paraplegia, while emphasizing the importance of careful patient selection and meticulous CSFD management to minimize potential complications [8].

Wong et al. analyzed strategies for preventing spinal cord injury in thoracic endovascular aortic repair [14]. The review focused on CSFD and discussed its potential utility, particularly for high-risk patients [14]. The optimal CSF pressure target during drainage, below 10 mmHg, was also discussed. Other strategies used in open surgical repair, such as maintaining spinal cord perfusion pressure and minimizing aortic occlusion duration, were briefly mentioned, but their applicability and effectiveness in TEVAR were not extensively explored [14].

Khan et al. demonstrated that CSFD significantly reduced the risk of SCI following TAAA repair [5]. The pooled analysis showed a nearly 50% reduction in SCI with CSFD [5]. The protective effect was more pronounced for early SCI but not statistically significant for late SCI [5]. The study concluded that CSFD could be an effective strategy, cautioned about potential complications, and emphasized the need for careful drain management [5]. However, the optimal drainage parameters and patient selection criteria were not adequately defined [5].

Batubara et al. discussed spinal cord ischemia prevention in the context of left subclavian artery revascularization during thoracic endovascular aortic repair [18]. The authors showed that LSA revascularization is associated with a statistically significant reduction in the risk of several ischemic complications [18]. Specifically, LSA revascularization significantly lowers the risk of stroke (OR 0.41), spinal cord ischemia (0.34), and left arm ischemia (0.22) [18]. However, the optimal revascularization techniques and patient selection criteria still warrant further investigation [18].

The role of prophylactic CSFD in preventing SCI after thoracic endovascular aortic repair was also examined by Zhang et al. [19]. Their review compared the use of prophylactic CSFD (placed before any signs of SCI) versus selective CSFD (inserted only after SCI symptoms appear) [19]. The use or not of prophylactic CSFD did not significantly affect the overall incidence of spinal cord injury (*p* = 0.51) in both the aortic aneurysm (*p* = 0.76) and aortic dissection subgroups (*p* = 0.70) [19]. However, the authors also acknowledge the limitations of the available evidence, including the utilized studies’ heterogeneity and the potential for publication bias [19].

Pini et al. found that the pooled SCI rate was lower for staged procedures compared to non-staged procedures (9% vs. 18%, respectively; *p* = 0.02), without significant difference in SCI rates between procedures staged over 1 month apart and those staged under 1 month apart [20]. While the staged approach showed a benefit in reducing SCI, the review also reported an inter-stage mortality rate of 1.6%, highlighting the potential risks associated with the staged approach [20]. In addition, the authors reported a similar pooled SCI rate of approximately 10% for both prophylactic and symptomatic CSFD, suggesting that prophylactic CSFD might not have offered a significant advantage in preventing SCI after TAAA-ER [20]. Finally, the authors commented that factors beyond hemodynamic changes, such as atheroembolization from a “shaggy aorta,” could also have contributed to SCI, and that focusing solely on CSFD might not have addressed all potential causes of SCI [20].

Chen et al. (2023) directly compared SCI rates in patients undergoing thoracic endovascular aortic repair with and without prophylactic CSFD [21]. The meta-analysis found no statistically significant difference in SCI rates between patients who received prophylactic CSFD and those who did not (OR 1.34, 95% CI 0.88–2.04, *p* = 0.17). These findings suggest that routine prophylactic CSFD may not offer a substantial benefit in reducing SCI risk for all TEVAR patients [21]. The study found no statistically significant difference in either transient or permanent SCI rates between the CSFD and non-CSFD groups [21]. The review also conducted subgroup analyses based on factors such as the type of aortic pathology and the CSFD strategy employed, yet these analyses likewise failed to reveal any significant differences in SCI rates [21].

Similarly, Frankort et al. incorporated twenty-eight observational, retrospective studies into their meta-analysis, involving a total of 4814 patients [7]. No significant reduction in spinal cord injury (SCI) was observed with the use of cerebrospinal fluid (CSF) drainage (OR 0.67, 95% CI 0.29–1.55, *p* = 0.35) [7]. The authors concluded that the placement of pre-operative CSF drainage did not correlate with a positive outcome in terms of SCI rates during endovascular repairs of thoraco-abdominal aortic aneurysms and descending thoracic aortic aneurysms (DTAA), but given the low quality of evidence, a definitive recommendation for the pre-operative use of CSF drainage placement could not be established [7].

According to Spinella et al.’s analysis of 53 studies involving 3095 patients, both the staged approach with reperfusion branches and the staged sequential approach with positioning of the thoracic component alternatives were associated with lower SCI risk, with type latter showing greater reduction, though less pronounced in older patients [22]. Additionally, the absence of cerebrospinal fluid, larger aortic diameter, and smaller aneurysm extent were associated with lower SCI risk [22]. Thus, a staged endovascular treatment, based on the patient’s anatomy and endovascular repair feasibility criteria, may provide significant advantages over single-step treatment in lowering the risk of spinal cord injury, irrespective of the reperfusion method employed [22].

Alzghari et al. discussed a couple of strategies for preventing SCI in the context of aortic repair, focusing on the use of prophylactic CSFD [2]. They suggested that CSFD can lower SCI rates after open TAAA repair, with less clear evidence of its effectiveness in TEVAR [2]. Additionally, the review states that maintaining adequate spinal cord perfusion is crucial for preventing SCI without delving into strategies to maintain mean arterial pressure within a specific target range and avoid hypotension. Finally, their study emphasizes the complexity of SCI prevention after aortic repair and the need for a multi-faceted approach [2].

Muston et al. discussed protective measures to reduce the risk of SCI during TAAA repair [3]. The authors state that most studies utilized spinal cord protection methods beyond induced hypothermia and rewarming, such as staging operations to allow spinal vasculature repair, and CSFD to lower pressure around the spinal cord [3]. Temporary aneurysm sac perfusion (TASP) was also used to maintain blood flow during surgery, while minimally invasive segmental spinal artery coil embolization (MISSACE) was less frequently employed [3].

Leone et al. summarized the complications associated with CSFD. At the same time, the authors recorded important details in the adopted CSFD protocols, depicting the heterogeneity in the clinical use of CSFD for SCI protection [24]. According to their review, the drains were placed using either anatomical landmarks or fluoroscopy guidance [24]. In most cases, the type of drain was not specified, but in two studies, the authors used Liquogard. Drains targeted either a specific pressure (10–12 mmHg) or a fixed CSF-flow rate (5–10 mL/h) [24]. In some studies, the drain was intermittently opened for 15 min every hour, allowing for a maximal CSF drainage of 20 mL [24].

Malloy et al.’s systematic review highlighted the variability in the indications and use of CSFD and the absence of a standard protocol [17]. Some studies indicated CSFD as a prophylactic measure for all patients, others in patients undergoing TEVAR, and the remaining primarily used it only in high-risk, selected patients [17]. Their review stated that the ideal timing, duration, and drainage parameters to maximize the benefits of CSFD remain unestablished, necessitating further studies in order to define an optimal CSFD protocol [17].

Zheng et al. were also among those who aimed to determine whether prophylactic use of CSFD contributes to a lower rate of SCI (SCI) after thoracic endovascular aortic repair (TEVAR) for Type B aortic dissection (TBAD) [6]. Based on a total of 34 studies involving 2749 patients, once again, the authors reported no reduction in the occurrence of permanent SCI with poutine or selective CSFD, and no difference in overall mortality between the two groups [6]. It is worth noting that the definition of “selective” CSFD was used inconsistently throughout the review, as it has been interchangeably used to refer to prophylactic drainage only in high-risk patients and drains placed after SCI [6].

According to Lella et al. CSFD, either routinely employed or in selected high-risk patients, was the most common measure to mitigate SCI after aortic repair [9]. High-risk patients included patients with longer aortic coverage, prior aortic surgery, and coverage of the subclavian and hypogastric vessels [9]. CSFD was set to a set pressure of 10 mmHg and 0 mmHg in asymptomatic and symptomatic patients [9]. Once again, the importance of TASP and MISAGE was shown, but the supporting evidence was scarce [9]. Regarding the perioperative care measures, the authors noticed an increased interest in optimizing hemodynamic flow parameters, including mean arterial blood pressure, as well as hemoglobin concentration and oxygen delivery [9]. The arterial pressure was maintained above 90 mmHg by either withholding antihypertensive medications or rarely using vasopressors, and the hemoglobin was kept above 10 g/dL [9]. Systemic or intrathecal steroids and intravenous naloxone were reported in a couple of studies without robust data on their safety and effectiveness [9].

### 3.7. Complications Associated with CSFD (Q5)

Seven studies reported complications associated with CSFD. Eight studies reported complications associated with CSFD. Khan et al. reported complications of CSFD during TAAA repair, including two patients requiring external ventricular drains, four experiencing intracranial hypotension, two instances of catheter occlusion or dislodgement, and one case of persistent cerebrospinal fluid leak requiring an epidural blood patch [5]. However, the exact complication rates could not be determined [5].

According to Zhang et al., the complications of CSFD included 1 case of subarachnoid hemorrhage, 1 case of epidural hematoma, 4 cases of intracranial hypotension, and 12 cases of headache, out of a total of 435 patients [19]. However, their review notes that the incidence of other complications, such as CSF leakage, infection, and entrapped drain, was not reported, so their specific rates remain unknown [19].

According to Chen et al., several additional complications were reported after prophylactic CSFD, including spinal headache (4.3%), meningitis (0.6%), CSF leak requiring re-intervention (0.7%), insertion site bleeding (0.7%), retained catheter tip (0.7%), epidural or spinal hematoma (0.9%), intracranial or subdural hemorrhage (0.8%), and significant paraparesis or paraplegia not attributable to ischemia (0.8%) [21]. This review also highlights two drain-related deaths following large intracranial bleeds, emphasizing the potential severity of CSFD complications [21]. The authors suggest that a larger total volume drained could be a risk factor for intracranial hemorrhage [21].

Frankort et al. attempted to quantify the incidence of complications associated with CSFD [7]. Their review estimated an overall complication rate of 13.6%, with a random-effects model estimate of 10% and a 3.4% rate in low-risk bias studies [7]. However, the review did not provide details on specific complication types due to limited reporting in the source studies [7].

Alzghari et al. categorized the complications of CSFD into three groups: severe (1.95%, including subdural hematoma and intracranial hemorrhage), moderate (0.38%, including spinal headache), and minor (1.81%, including puncture site bleeding) [2]. The authors noted that the rates were pooled estimates that may have varied across studies [2].

A review by Zheng et al. focuses on the impact of prophylactic CSFD on outcomes like SCI and mortality rather than comprehensively analyzing CSFD-related complications [6]. While the review does not quantify complication rates, it acknowledges the potential for adverse events [6]. The discussion mentions complications like intracranial hypotension and meningitis, but the included studies did not consistently report these, limiting detailed analysis [6].

According to the review by Malloy et al., the pertinent literature commonly reported low-severity complications associated with CSFD, such as spinal headache and puncture site pain, with a reported incidence rate as high as 23% [17]. While fewer studies detailed complications requiring intervention, the available evidence suggests that more serious issues, including spinal hematomas, an entrapped drain, and cerebrospinal fluid leaks, did occur in some cases [17]. Importantly, no studies reported persistent morbidity or mortality directly attributed to CSFD procedures [17].

Leone et al. identified seven CSFD-related deaths among 365 patients in three publications [24]. The authors estimated the crude mortality rate and the random effects model mortality to be as high as 1.9% and 1.4%, respectively, both of which are non-negligible figures [24]. Notably, all deaths occurred after massive intracranial hemorrhage during hospitalization or within 30 days after surgery [24].

### 3.8. Treatment (Q6)

None of the studies reviewed directly focused on summarizing the evidence on managing postoperative SCI. Similarly, no review addressed the management of delayed SCI. However, two studies did discuss relevant aspects of SCI treatment as a secondary aim.

Postoperative SCI management, according to Lella et al., focuses on prompt diagnosis and treatment [9]. Strategies for managing postoperative SCI include hemodynamic optimization, which involves maintaining adequate mean arterial pressure, often above 80 mmHg or higher, depending on patient-specific factors [9]. Permissive hypertension may be employed, in the sense of withholding antihypertensive medication [9]. Continuing or initiating CSF drainage to reduce pressure on the spinal cord is a common practice, with target pressures generally below 10–15 mmHg [9]. Surgical intervention, such as decompression or revascularization, may be considered in cases of persistent or worsening neurological deficits [9]. Supportive care, which includes maintaining adequate oxygenation, managing fluid and electrolyte balance, and providing appropriate pain control, is also crucial. Regular neurological assessments are essential to monitor for changes in neurological status and guide treatment decisions [9]. The review emphasizes the importance of a multidisciplinary approach involving vascular surgeons, neurologists, and critical care specialists. It also highlights the need for further research to optimize postoperative SCI management strategies [9].

Pini et al. focused on the occurrence of SCI after endovascular repair of TAAA and mentioned certain measures, such as pharmacologic blood pressure support, direct usage of CSFD, and intensive physiotherapy, as being clinically useful [20]. Significant clinical amelioration was observed in selected patients after stenting a stenotic hypogastric artery [20].

### 3.9. Prognosis (Q7)

Most of the systematic reviews mentioned the overall mortality rates in the context of open surgical or endovascular repair but did not primarily address mortality specifically associated with SCI. Tanaka et al. reported that the total mortality rate across the included studies was 6.9% (54/782 patients) [15]. Likewise, Algzhari et al. (2023) distinguish between operative mortality, which is death during the initial hospital stay or within 30 days of the operation if the stay was shorter, in up to 6.2% of cases, and late mortality, defined as death occurring after the initial hospital stay or after 30 days post-operation, without a single pooled late mortality rate but presenting data for different follow-up periods [2]. Muston et al. added that the 36-month survival rates were as follows: the hybrid group had the highest survival at 88.7%, the open group had a survival of 61.7%, while the endovascular group had insufficient data for meaningful interpretation [3].

Wong et al. were among those to comment on the negative impact of SCI on medium-term survival [14]. The review references a single study which found that perioperative SCI after thoracic aortic interventions is associated with impaired medium-term survival [14]. Once again, the review does not provide precise mortality rates attributable to SCI [14]. Likewise, Lella et al. noted that patients with permanent paraplegia due to SCI were characterized by a poorer prognosis and had a mortality rate as high as 75% for DTA, and 44% for TAAA within the first year after surgery [9].

On the other hand, Moulakakis et al. focused on 25 cases with SCI from 18 studies regarding neurological improvement [1]. Their review included patients with variable presentation, ranging from mild sensory deficits to complete paraplegia, and mentions that common symptoms include motor weakness (paraparesis or paraplegia), sensory disturbances, and bowel or bladder dysfunction [1]. The specific neurological deficits depended on the level and extent of spinal cord involvement and are indicative of the definition of SCI variability [1]. In half of these cases, patients exhibited only modest improvement at follow-up. In a quarter of the cases, no improvement was observed, while a quarter of cases demonstrated near-complete recovery [1].

### 3.10. Results of Quality Appraisal

**Figure 2:**
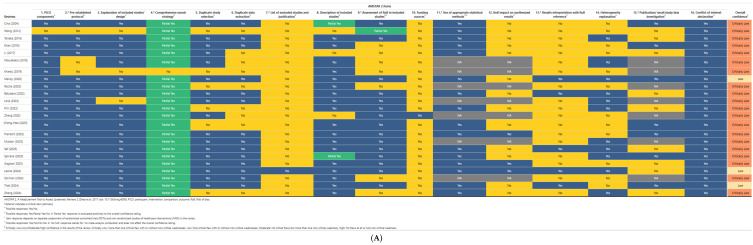

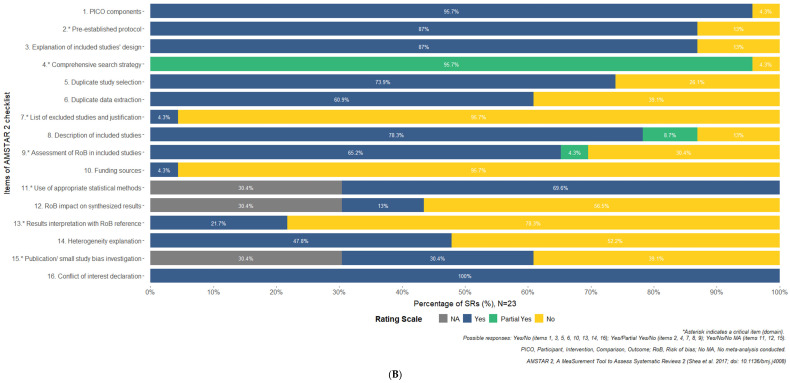
AMSTAR-2 grading for each review study individually (**A**) and for the whole dataset (**B**) [1,2,3,4,5,6,7,8,9,11,14,15,16,17,18,19,20,21,22,23,24,25,26].

Of the studies, twenty-three were rated as being “critically low” and two as “low” quality according to AMSTAR-2. It is worth noting that no study reported the list of the excluded records except one [7], and no study considered the role of funding or the risk of bias while discussing their results (Figure 2). Furthermore, the clinical heterogeneity of the available data precluded performing an umbrella meta-analysis that could help us estimate the risk of publication bias.

The eligible reviews and meta-analyses of our study were largely characterized by a high risk of bias, frequent inconsistencies or conflicting findings, and a lack of direct evidence on numerous topics (Table 5). It is also notable that only a few studies controlled for potential moderators. Thus, according to the GRADE recommendations, the overall quality of evidence was moderate for Q1, Q4, and Q5, low for Q3, and very low for Q2, Q6, and Q7, suggesting that further research is still needed to better characterize the underlying pathogenesis, methods for early SCI detection, and treatment strategies for perioperative SCI (Table 6).

## 4. Discussion

### 4.1. Overview of Our Findings

This paper includes systematic reviews and studies that provide a comprehensive overview of the current evidence on diagnosing, preventing, and managing SCI in the context of thoracic and thoraco-abdominal aortic interventions. SCI has been found to occur with a reported incidence ranging from 1.4% to 10% for open repair and 3% to 10% for endovascular procedures. Permanent paraplegia remains in less than 5% of the cases. Among the risk factors of SCI, extensive aortic coverage, poor collateral blood perfusion, reoperation, and hemodynamic instability have been identified (Q1). The reviewed studies also highlight the effectiveness of various interventions, such as CSFD, pharmacologic blood pressure support, and selective revascularization, in preventing postoperative issues (Q4). Finally, CSFD seems to have non-negligible morbidity and mortality, and its use should weigh the benefits against the potential risks (Q7).

### 4.2. Prevention and Treatment of SCI in the Guidelines

Several clinical guidelines were published in 2024, including the “2024 ESC Guidelines for the management of peripheral arterial and aortic diseases”, the “European Society for Vascular Surgery (ESVS) 2024 Clinical Practice Guidelines on the Management of Abdominal Aorto-Iliac Artery Aneurysms”, and the “AO Spine & Praxis Spinal Cord Institute Guidelines for the Management of Acute Spinal Cord Injury”. These guidelines provide comprehensive recommendations to prevent and manage spinal cord injury [27,28].

The ESVS recommendations support the assertion that SCI is more common after the open or endovascular repair of TAAAs, types I, II, and III [29]. The endovascular repair of complex AAAs may triple the SCI risk compared to open surgery, although recent studies show varying incidences [29]. Preventive strategies include staging the procedure, maintaining high blood pressure and oxygenation, preserving collateral circulation, CSFD, and neuromonitoring [29]. Prophylactic CSFD is proven for open TAAA repair but lacks evidence for complex AAA EVAR [29]. Due to potential complications, routine prophylactic CSFD is not recommended for complex AAA repair, but may be considered in high-risk patients [29]. Rapid post-operative extubation for neurological assessment is desirable, and a rescue drainage policy is often preferred over prophylactic drainage [29].

According to the ESC guidelines, SCI is reported in 11% to 15% of cases, and is often linked to the severity of aortic pathology [30]. However, thoracic endovascular aortic repair has been associated with an increased risk of SCI, underscoring the need for a prudent approach to revascularization strategies [30]. This may involve the prior surgical or concurrent endovascular revascularization of the left subclavian artery in elective settings [30]. The guidelines do not comment on neuromonitoring or the use of CSFD and provide no treatment strategy in the event of SCI [30].

AO Spine defines intraoperative spinal cord injury as a new or worsening neurological deficit due to spinal cord dysfunction diagnosed during surgery, using neuromonitoring, wake-up tests, or immediate post-operative clinical assessment [31]. The reported frequency of intraoperative SCI varies widely from 0% to 61%, with risk factors including older age, male sex, cardiovascular disease, severe myelopathy, blood loss, osteotomy, and spinal deformities [31]. Conversely, better preoperative neurological status and intraoperative neuromonitoring are associated with decreased ISCI risk [31]. Multimodal monitoring can achieve high sensitivity (83.5%) and specificity (93.8%) [32]. The authors recommend a comprehensive checklist of surgical, technical, and anesthetic measures to prevent and manage ISCI, emphasizing communication between surgeons and anesthesiologists [32]. In the event of persistent neurophysiologic changes, options include steroids, consulting colleagues, wake-up tests, and potentially aborting the procedure [32]. Post-operative management involves close monitoring, steroids, blood pressure control, and further imaging [32]. AO Spine also recommended maintaining the mean arterial blood pressure between 75 and 80 mmHg, but not exceeding 90–95 mmHg, in order to optimize spinal cord perfusion in patients with acute traumatic SCI [28]. This recommendation is for a time period of at least three to seven days [28]. However, the attending physician should use their discretion in selecting the appropriate vasopressor or inotrope to achieve the target mean arterial pressure goals in these patients [28]. The guidelines do not provide any commentary on the use of CSFD, and they also lack a clear definition of the optimal steroid treatment and its recommended dosage.

### 4.3. Evidence Gaps and Future Perspectives

The current umbrella review identified five important gaps in the literature. Firstly, the literature does not consistently define and report SCI. Most reviews did not distinguish between permanent and temporary neurological deficits, which could have important implications for patient outcomes and management. Minor motor and sensory deficits were not registered. The absence of consistent definitions extends to the terms “temporary and “permanent” neurological deficit, a fact which adds to the complexity in understanding this disabling complication. A detailed, multifaceted definition that considers severity, timing, and other factors like neurological level should be broadly used and supported by the appropriate guideline bodies. Moreover, many of the reviewed studies did not assess long-term neurologic outcomes beyond the immediate perioperative period.

Secondly, all risk factors point towards an ischemic etiology for SCI, with the final pathway likely being, most likely, the hypoperfusion of the spinal cord during aortic surgery (Q2). The surgical manipulation, aortic cross-clamping, and coverage of the involved segmental arteries during repair can compromise spinal cord perfusion, leading to ischemia and subsequent neurological deficits. Nevertheless, the pathogenesis seems to be multifactorial and incompletely understood, implicating factors like inadequate revascularization, elevated CSF pressure, and spinal cord compression from hematomas to be less studied. Likewise, the molecular pathogenetic pathways, including spinal cord edema, neuroinflammation, excitotoxicity, spasm of the microcirculation, mitochondrial dysfunction, oxidative stress, and apoptosis, are not fully characterized. Moreover, it is not clear if the underlying mechanisms of delayed SCI are the same as in acute/peri-operative injury. Understanding the pathogenetic mechanisms is critical in the development of effective targeted preventive and therapeutic interventions.

Thirdly, no established method exists to monitor or measure spinal cord perfusion intraoperatively to guide surgical and anesthetic management (Q3). Modern techniques, like somatosensory and motor-evoked potentials, remain an established method in spine surgery. However, their accuracy in aortic repair surgery could be affected by several factors, such as hypothermia, cardiopulmonary bypass, and drug selection during anesthesia. Therefore, further comprehending the limitations and optimizing the use of neuromonitoring strategies is of utmost importance. Other methods, like CSF biochemistry and NIRS, seem to be less promising, at least in the near future.

Fourthly, a lack of consensus on the optimal treatment strategies is observed, althrough this is not to say that there are no treatment methods at all (Q6). It is clear that endovascular repair is associated with significantly less risk for SCI. Likewise, staged procedures, F/B endovascular aortic repair, and LSA revascularization have shown protective effects against SCI [33,34]. Empasis is also placed on the optimal aneurysm selection, due to inherent differences in the associated SCI risk. However, many of the implemented measures are, in fact, extensions of the preventive measures during postoperative care. Among these are the use of CSFD, blood pressure augmentation, hypothermia, pharmacologic agents, and stimulation strategies [35]. Moreover, the target CSFD remains ill-defined, with significant differences among studies. The role of steroids is also unclear. In spinal cord trauma, early high-dose methylprednisolone has been advocated for a limited period of 24 h in highly selected young patients without any underlying comorbidities, but its utility in SCI associated with aortic procedures is not proven. Likewise, specific vasodilators or other neuroprotective medications with documented benefits after cerebral aneurysmal vasospasm, like nimodipine, milrinone, or fasudil, warrant further investigation. It is of note that the “ISCOPE” trial by Papadopoulos et al. demonstrated that neurologic recovery may be more closely tied to spinal cord perfusion pressure (SCPP) than to mean arterial pressure. Future guidelines may include SCPP monitoring and management recommendations as new evidence emerges, with stratification for early and delayed SCI.

Finally, the impact of SCI on long-term functional outcomes and quality of life remains poorly characterized. While the available studies provide mortality rates, they do not systematically report on SCI-related parameters. These figures are important to guide patients and their families in the complex decision-making process surrounding the risks and benefits of thoracic or thoraco-abdominal aortic interventions. Moreover, accurate data would support future research endeavors, cost-effectiveness analyses, and healthcare policy decisions. In addition, future studies need to address several functional patient-reported data, including quality of life, overall patient functioning, psychological distress, and social parameters.

### 4.4. Ongoing Trials

Numerous ongoing clinical trials are poised to yield valuable insights regarding the prevention and management of SCI in the context of TAAA repair procedures. The clinical trial denoted as NCT04941157 serves as a pilot study, specifically aiming to evaluate the feasibility of conducting a larger randomized controlled trial that contrasts the prophylactic versus selective placement of CSFD for SCI prevention in patients undergoing endovascular TAAA repair. This investigation seeks to ascertain whether the implementation of prophylactic CSF drains, as part of a comprehensive SCI prevention protocol, effectively diminishes the incidence of SCI compared to a strategy of placing drains exclusively upon the manifestation of SCI.

Similarly, the SINATRA trial (NCT03074487) is focused on exploring methodologies for the early detection of SCI subsequent to open surgical interventions for TAAA. This study aims to assess the accuracy and feasibility of alternative neurophysiological assessments for the postoperative detection of SCI in sedated or partially sedated patients. Among the diagnostic modalities being evaluated are long loop reflexes (LLR) utilizing F-waves and paraspinal muscle oxygenation metrics measured through near-infrared spectroscopy (NIRS).

Conversely, the trial labeled NCT05195905 investigates the application of physician-modified endografts (PMEG) in treating pararenal and thoracoabdominal aortic aneurysms. This research intends to enroll approximately 30 participants aged 18 years and older. The primary outcomes to be measured encompass the incidence of major adverse events within 30 days post-procedure and the success of the treatment at the 12-month follow-up. Secondary outcomes include mortality rates, incidences of major adverse events, occurrences of spinal cord ischemia, treatment success, and the rate of freedom from secondary interventions.

The PAPA-ARTiS trial (NCT03434314) is designed to investigate the potential reduction in paraplegia and mortality risk associated with the minimally invasive staged aortic coiling and embolization (MISACE) procedure in patients undergoing thoracoabdominal aortic aneurysm repair. This multinational, prospective, open-label, two-arm randomized controlled trial compares two distinct treatment strategies: TAAA repair conducted with versus without MISACE pre-treatment, the latter of which involves the staged embolization of segmental arteries prior to aneurysm repair in an effort to enhance collateral blood flow to the spinal cord.

Lastly, the CASPER trial represents a multicenter initiative aimed at enrolling 100 patients diagnosed with acute traumatic cervical and thoracic SCI to explore the influence of spinal cord perfusion pressure (SCPP) management on neurological outcomes. Patients will have a lumbar intrathecal catheter placed within 24 h post injury for the purposes of intrathecal pressure (ITP) measurement and CSF sampling. The primary objective of this study is to ascertain whether the maintenance of an SCPP ≥ 65 mmHg yields improved neurological recovery outcomes in comparison to conventional hemodynamic management practices. Additionally, the trial will investigate the feasibility of CSF drainage to mitigate ITP while assessing the complications associated with intrathecal catheter insertion and CSF drainage in this specific patient demographic.

### 4.5. Key Challenges in Studying Spinal Cord Injury

The rarity of SCI and the inherent difficulties in conducting large-scale clinical trials pose significant barriers to advancing research in this field. Multidisciplinary collaboration among vascular surgeons, anesthesiologists, critical care physicians, neurosurgeons, neurologists, and rehabilitation specialists is essential to comprehensively investigate the various aspects of this complex clinical condition. Securing adequate funding and overcoming the inherent heterogeneity among SCI cases further contribute to the slow progress in this area of study. Additionally, the use of diverse definitions and outcome measures in the available literature makes it challenging to synthesize the evidence and draw firm conclusions. Despite these limitations, the past decade has witnessed a substantial increase in the volume of published research on SCI associated with aortic surgery. Establishing standardized registries with well-defined parameters would be instrumental in advancing this field of study.

### 4.6. The Role of CSF Drainage

The current review identifies prophylactic cerebrospinal fluid (CSF) drainage as a commonly used technique in open elective TAAA repairs to preserve spinal cord perfusion, aiming to maintain intracranial pressure below central venous pressure or 10 mmHg. Drainage rates typically fall within the range of 5 to 15 milliliters per hour (mL/h), with some centers opting for slightly higher rates, up to 20 mL/h, during the intraoperative period. Postoperatively, many protocols recommend a more conservative drainage rate, often not exceeding 10 mL/h. The total volume of CSF drained can also vary significantly, with some protocols specifying maximum volumes, such as no more than 25 mL in a four-hour period or 150 mL within a 24 h window. The overall duration of CSF drainage typically extends for 24 to 72 h following surgery in patients who do not exhibit any neurological deficits. However, in cases where SCI symptoms develop, the drainage period may be prolonged, sometimes lasting up to 5 to 7 days. This approach is often supplemented by strategies to ensure adequate blood pressure and cardiac output. However, the effectiveness of CSF drainage in thoracic endovascular aortic repair (TEVAR) is debated, with some centers recommending it for high-risk patients while others advocate for selective use only. Although intended to prevent spinal cord ischemia, the procedure carries risks, including headaches and infections, necessitating careful patient selection and monitoring. To optimize the use of prophylactic CSF drainage, further investigation into specific drainage parameters from medical associations and the detailed exploration of patient selection criteria is crucial, along with recommendations for future randomized controlled trials to provide more definitive evidence.

### 4.7. The Role of Neuromonitoring

The adjustment for confounding variables such as operation duration and aortic coverage, disturbances in oxygenation and core body temperature is paramount for the accurate interpretation of intraoperative recordings in abdominal aortic repair surgery. Within this context, multimodal neuromonitoring—specifically the integration of MEPs and SSEPs—emerges as a significant tool for the intraoperative evaluation of spinal cord functionality during surgery. This combinatorial approach is characterized by enhanced sensitivity and specificity, which may facilitate the improved identification of risks associated with SCI. Nevertheless, the literature surrounding the definitive impact of neuromonitoring on the reduction in paraplegia rates presents conflicting evidence, likely attributable to variability in monitoring protocols and the influence of multiple external factors. Therefore, it is essential to embed neuromonitoring within a comprehensive strategy for SCI prevention, which should include the implementation of CSFD, distal aortic perfusion, and the selective re-implantation of intercostal arteries. To elucidate the role of neuromonitoring and refine its application in enhancing neurological outcomes for patients undergoing abdominal aortic repair, further empirical research, particularly well-structured randomized controlled trials that adequately account for possible confounders, is warranted. In addition, intraoperative neuromonitoring might help identify patients at risk of developing delayed SCI. Given the dynamic nature of both open and endovascular surgical techniques, a continuous reassessment of optimal neuromonitoring strategies is necessary, alongside the recognition that the presence of proficient multidisciplinary teams is critical for the successful adoption and interpretation of these advanced monitoring techniques.

### 4.8. Limitations

The current umbrella review is limited by the shortcomings of primary and secondary studies. Firstly, it is based on small-sized, single-center, retrospective studies, which carry a risk of bias. Secondly, the reporting clarity of the secondary reviews and meta-analyses is often suboptimal, limiting the ability to assess methodological quality. Thirdly, there is substantial clinical heterogeneity among the included studies in terms of definitions of SCI, patient population, surgical procedures, and reported outcomes. Fourthly, the limited available evidence precludes the stratification of the findings according to the implemented technique, patients’ age and gender, aneurysm extent, and studies from low- or middle-income countries. Finally, as in every umbrella review, there is an obvious bias against newer techniques and devices.

## 5. Conclusions

SCI remains a devastating complication after aortic repair surgery with a variable rate depending on several factors, including the type of repair and the adopted prophylactic measures. To effectively address the existing gaps in our understanding of SCI related to thoracic and thoraco-abdominal aortic procedures, future research should prioritize standardizing definitions of SCI and associated neurological deficits to ensure consistency across studies, thereby enhancing communication and collaboration within the research community. Furthermore, a deeper examination of the pathogenic mechanisms of SCI at the cellular and molecular levels is necessary to identify potential therapeutic targets. Optimizing neuromonitoring techniques through multimodal approaches could significantly improve the intraoperative and early postoperative detection of SCI. Conducting high-quality studies, particularly randomized controlled trials, is vital for assessing the effectiveness of preventive strategies such as CSFD, staged procedures, and meticulous hemodynamic management, alongside establishing evidence-based treatment protocols that explore pharmacological interventions and surgical decompression strategies. Identifying high-risk patients who may be susceptible to CSFD-related complications and developing strategies to mitigate these risks is crucial, as is a thorough assessment of the long-term morbidity and mortality linked to SCI after aortic repair, which will provide valuable insights for patient management and prognostication. By addressing these research priorities, we can significantly enhance our ability to prevent, diagnose, and manage SCI, leading to improved outcomes for patients undergoing aortic surgery.

## Figures and Tables

**Table 1:** The research questions summarized in the PICOT format.

Research Question	Patient	Intervention	Comparator	Outcome	Time
Q1	Patients undergoing aortic surgery	Risk factors related to SCI following surgery	None or standard aortic surgery	Incidence and risk factors for SCI	ANY
Q2	Patients experiencing SCI post-aortic surgery	Mechanisms leading to SCI	Standard aortic surgery patients without SC	Understanding of pathogenesis in SCI after surgery	ANY
Q3	Patients at risk for SCI post-aortic surgery	Early diagnostic methods	Standard diagnostic methods or no early diagnostic approach	Diagnostic accuracy, timeliness, and effectiveness in detecting SC	Intraoperative and early postoperative periods
Q4	Patients undergoing aortic surgery at risk for SCI	Preventive interventions	Standard care without specific preventive measures	Incidence of SCI, early detection, and neurological outcomes	Intraoperative period, early post surgery
Q5	Patients undergoing aortic surgery at risk for SCI	Presence of complications linked to CSFD	No CSFD or standard care	Incidence of complications linked to CSFD	Early and late postoperative periods
Q6	Patients with SCI post-aortic surgery	Treatment options	No treatment or standard management protocols	Recovery of neurological function and reduction in SCI severity	Immediate postoperative to long-term follow-up
Q7	Patients with SCI following aortic surgery	Factors influencing prognosis	No SCI or standard aortic surgery without complication	Functional recovery, quality of life, mortality rates	Short-term to long-term follow-up

**Table 2:** Basic characteristics of the twenty-three eligible studies.

Authors	SD	Databases	Period	Patients	Intervention	Comparator	Outcomes	Moderators	Quality Assessment	Studies (Patients)	Study Question (s)
Cina (2004) [8]	MA	Ovid software	Until June 2002	Patients requiring TAAA repair	Prophylactic CSFD	No prophylactic CSFD	SCI and complications	Subgroup analysis for study design	Jadad three-item scale	8 (1143)	Q1, Q4
Wong (2012) [14]	MA	PubMed, the Cochrane Library, and Conference abstracts	2000–2011	Patients undergoing TEVAR	Prophylactic CSFD	No prophylactic CSFD	SCI	(-)	Downs and Black checklist	46 (4936)	Q1, Q5
Tanaka (2016) [15]	MA	MEDLINE, the Cochrane Central Register, EMBASE, CINAHAL, and the Japanese Central Review of Medicine	Until September 1st 2015	Patients requiring TAAA repair	TAAA surgery with MEPs	(-)	Sensitivity and specificity for detecting SCI	Anesthesia, and surgery details as moderators for MEPs accuracy; subgroup for several cut-off values	QUADAS-2	19 (-)	Q3
Khan (2016) [5]	MA	Clinicaltrials.gov, Cochrane Library, PubMed/MEDLINE, and Scopus	Until August 2015	Patients requiring TAAA repair	CSFD	W/O or selective CSFD	SCI	Early vs. late SCI	Jadad and NOS	10 (2013)	Q1, Q4, Q5
Li (2017) [11]	MA	PubMed, EMBASE, Cochrane Library, Web of Science, and ScienceDirect	(-)	Patients with TBAD requiring surgery	TEVAR	OS, BMT	30-day/in-hospital mortality and morbidity, including paraplegia	(-)		16 (10,307)	Q1
Moulakakis (2018) [1]	SR	PubMed and Scopus	Until December 2017	Patients with infrarenal AAA	Elective endovascular repair	(-)	SCI, including its incidence, risk factors, clinical presentation, and outcomes	Patient characteristics, procedural factors, and anatomical variations		18 (25)	Q1, Q7
Harky (2019) [16]	SR	PubMed, MEDLINE, EMBASE, Google Scholar, SCOPUS, and Cochrane	(-)	Patients requiring TAAA repair	(-)	(-)	SCI	(-)	(-)	15 (265)	Q3
Rocha (2020) [4]	MA	MEDLINE and EMBASE	January 2006 to March 201	Patients with thoracic aortic pathology	Open repair	Endovascular repair	In-hospital or 30-day mortality and morbidity, including SCI	Permanent vs. overall	Checklist	71 (-)	Q1, Q2
Malloy (2020) [17]	SR	PubMed/ MEDLINE, Scopus, Ovid, Cochrane, and EMBASE	1 January 2016 to 17 December 2018	Patients with aortic aneurysm requiring repair	TEVAR	(-)	SCI (overall vs. temporary)	Various CSF protocols	Joanna Briggs Institute checklist	8 (859)	Q1, Q4, Q5
Batubara (2022) [18]	MA	PubMed, Ovid Medline, and Cochrane	NR	Patients undergoing Thoracic EVAR	With LSAR	W/O LSAR	Ischemia, stroke, and SCI	(-)	NOS	22 (11,386)	Q4
Zhang (2022) [19]	MA	MEDLINE, EMBASE, and Cochrane	Until 1 April 2020	Patients undergoing TEVAR	Prophylactic CSFD	W/O or selective prophylactic CSFD	SCI, CSFD-related complications	Pathology (aneurysm vs. dissection); hybrid procedures	Downs and Black score	34 (3561)	Q1, Q4, Q5
Pini (2022) [20]	MA	PubMed, EMBASE, and Cochrane Database	Until 1 February 2021	Patients with TAAA	Endovascular repair	(-)	SCI	(1) TAAA extension; (2) overall vs. permanent SCI; (3) use of CSFD; (4) prophylactic vs. symptomatic; (5) staged vs. nonstaged approach	NOS	27 (2333)	Q1, Q4, Q6
Lella (2022) [9]	SR	Cochrane and PubMed	2012 to 2021	Patients with DTA and TAAA	Open or endovascular repair	(-)	SCI	Type and extent of aortic pathology, operative technique, SCI protection or mitigation strategies, and rates of overall and permanent SCI	NR	41 (-)	Q1, Q4, Q6
Cheng-Hao (2023) [21]	MA	Scopus, EMBASE, Medline, and Cochrane and Evidence-Based Medicine Reviews	Until September 2022	Patients undergoing TEVAR	Routine CSFD	Selective CSFD and no CSFD	SCI (any vs. permanent vs. transient) rate, complication rates, and operative outcomes	Immediate vs. delayed; transient vs. permanent; aneurysm vs. dissection; emergency vs. elective	Modified Institute of Health Economics scale	40 (4973)	Q1, Q4, Q5
Frankort (2023) [7]	MA	MEDLINE, EMBASE, and CINAHL	Until November 2022	Patients undergoing TEVAR	Prophylactic CSFD	W/O prophylactic CSFD	SCI (early onset of late), complications, and mortality	Risk of bias	NOS/GRADE	28 (4814)	Q1, Q4, Q5, Q7
Muston (2023) [3]	MA	EMBASE, PubMed, Scopus	Until 3 January 2023		Open group	Endovascular (TEVAR and F/B EVAR), and hybrid groups	SCI (permanent, in-hospital)	(-)	CNIHE tool	20 (924)	Q1, Q4
Spinella (2023) [22]	MA	PubMed/MEDLINE	Until November 2020	Patients with TAAA	Type A: single step	Type B: staged with reperfusion branches; Type C: staged with positioning of the thoracic component	SCI (transient and permanent)	Age, extent of the aneurysm, the diameter of the aneurysm, and the use of CSFD	(-)	53 (3095)	Q1, Q4
Sef (2023) [23]	SR	PubMed, EMBASE via Ovid, Cochrane library, and Clinical Trials Gov	Until December 2022	Patients undergoing open TAAA repair	Neuromonitoring methods MEPs, SSEPs, NIRS, and TCD		Mortality, SCI, and neurologic deficit	(-)	NOS	27 (3130)	Q1. Q2, Q3
Alzghari (2024) [2]	MA	Ovid MEDLINE, Ovid EMBASE, and the Cochrane Library	Until September 2022	Patients with DTA and TAAA	Open or endovascular repair	(-)	SCI, temporary SCI, operative mortality, long-term mortality, postoperative stroke, and CSFD-related complications	Subgroup analyses and multivariate analyses for a multitude of factors	NOS	239 (61,962)	Q1, Q4, Q5, Q7
Leone (2024) [24]	MA	MEDLINE, EMBASE, and Scopus	2000–2023	Patients undergoing F/B-EVAR	CSFD	(-)	CSFD-related mortality and morbidity	(-)	NOS	6 (730)	Q4, Q5
Zheng (2024) [6]	MA	PubMed, EMBASE, Web of Science, and Cochrane Library	Up to May 2023	Patients undergoing TEVAR (elective or emergency) for TBAD	Routine prophylactic CSFD	Selective prophylactic CSFD, W/O prophylactic CSFD	Permanent SCI, temporary SCI, CSFD-related complications, and 30-day mortality	With or W/O CSFD	Downs and Black score	34 (2749)	Q1, Q4, Q5
Thet (2024) [25]	SR	PubMed, MEDLINE via Ovid, EMBASE, Scopus, and Cochrane CENTRAL	1998–2024	Patients with TAAA	TEVAR or F/B-EVAR	(-)	SCI and early mortality	Permanent vs. transient, neuromonitoring (MEPs, SSEPs, NIRS)	NOS	11 (1069)	Q1, Q3, Q4
Soliman (2024) [26]	SR	PubMed, Scopus, and Google Scholar	1995–2022	Experimental animals or patients undergoing TAAA repair	Detection and monitoring of SCI using various monitoring techniques	(-)	SCI, SC-blood flow	(-)	NR	59 (-)	Q3

BMT, best medica treatment; CSF, cerebrospinal fluid; CSFD, cerebrospinal fluid drain; CT, computed tomography; CNIHE, Canadian National Institute of Health Economics; DTA, descending thoracic aorta; F/B-EVAR, fenestrated or branched endovascular repair; GRADE, Grading of Recommendations Assessment, Development and Evaluation; LSAR, left subclavian artery revascularization; MA, meta-analysis; MEPs, motor-evoked potentials; MICACE, minimally invasive segmental artery embolization; MRI, magnetic resonance imaging; NIRS, near-infrared spectroscopy; NOS, Newcastle Ottawa scale; OS, open surgery; SC, spinal cord; SCI, spinal cord ischemia; SD, study design; SR, systematic review; SSEPs, somatosensory evoked potentials; QUADAS-2, Quality Assessment of Diagnostic Accuracy Studies—2; TAAA, thoracoabdominal aortic aneurysm; TASP, temporary aneurysm sac perfusion; TBAD, Type B aortic dissection; TCD, transcranial Doppler; TEVAR, thoracic endovascular aortic repair; US, ultrasound; W/O, without.

**Table 3:** Incidence of spinal cord ischemia in the gathered studies.

Authors	Studies (Patients)	Overall	Transient	Permanent	Early	Late
Cina (2004) [8]	8 (1143)	33%	(-)	(-)		
Wong (2012) [14]	46 (4936)	3.47% (95% CI, 1.98–5.37%)				
Tanaka (2016) [15]	19 (-)	0 to 16.7%				
Khan (2016) [5]	10 (2013)	(-)				
Li (2017) [11]	16 (10,307)	0 to 50%				
Moulakakis (2018) [1]	18 (25)	1% (range: 0–8%)				
Harky (2019) [16]	15 (265)	(-)				
Rocha (2020) [4]	71 (-)	Endovascular: 13.5% (95% CI, 10.5–16.7%); Open: 7.4% (95% CI, 6.2–8.7%, *p* < 0.01)		Endovascular: 5.2% (95% CI, 3.8–6.7%); Open: 4.4% [95% CI, 3.3–5.6%, *p* = 0.39]		
Malloy (2020) [17]	8 (859)	0–17%		0–2%		
Batubara (2022) [18]	22(11,386)	2.5% (*n* = 283, R = 11,065)				
Zhang (2022) [19]	34 (3561)	Endovascular for aortic: 3.49% (95% CI, 0.23–6.76%); Endovascular for dissection: 3.20% (95% CI, 0.00–7.20%)				
Pini (2022) [20]	27 (2333)	Endovascular: 11% (95%CI, 8–15%)				
Lella (2022) [9]	41 (-)	Overall: 0–16%; Endovascular 0–35%; Open: 3.1–33.5%		Endovascular: 2–20.5%; Open: 1.11%		
Cheng-Hao (2023) [21]	40 (4973)	TEVAR: 3.5% (95% CI: 2.6–4.4%)			1.3% (95% CI: 0.7–1.8%)	1.9% (95% CI: 1.2–2.5%
Frankort (2023) [7]	28 (4814)	Endovascular: 5%, (95% CI 0–14%)				
Muston (2023) [3]	20 (924)	Overall: 5.4% (95%CI 5.1–5.8%); Open: 1.4% (95% CI, 1.3–1.5%); Hybrid: 3.2% (95% CI, 2.8–3.6%); Endovascular: 9.8% (95% CI, 9.2–10.4%)				
Sef (2023) [23]	27 (3130)				0% to 17.0%	1.3% to 12.0%
Alzghari (2024) [2]	239 (61,962)			Overall: 3.3% (95%CI 2.9–3.8%); Open 4.0% (95% CI, 3.3–4.8%); Endovascular: 2.9% (95% CI, 2.4–3.5%)		
Zheng (2024) [6]	34 (2749)		1.0% (95% CI, 0.00–1.0%)	2.0% (95% CI, 1.0–2.0)		
Thet (2024) [25]	11 (1069)	3.8 to 17.3%		2.7 to 5.8%		

**Table 4:** Summary table for the overall, permanent, and late spinal cord ischemia incidence according to the implemented approach.

Incidence	Technique	Range	Reference Studies
Overall	Open	1.4–33.5% (prevalent value around 10%)	Rocha, 2019 [4]; Lella, 2022 [9]; Muston, 2023 [3]
	Endovascular	0–35% (prevalent value around 3.5%)	Wong, 2012 [14]; Rocha, 2019 [4]; Pini, 2022 [20]; Zhang, 2022 [19]; Lella, 2022 [9]; Cheng-Hao, 2023 [21]; Frankort, 2023 [7]; Muston, 2023 [3]
	Not defined	3.8–33%	Cina, 2004 [8]; Lella, 2022 [9]; Muston, 2023 [3]
Permanent	Open	3.3–11%	Rocha, 2019 [4]; Alzghari, 2024 [2]
	Endovascular	2.9–6.7%	Rocha, 2019 [4]; Lella, 2022 [9]; Alzghari, 2024 [2]
	Not defined	2–7–5.8%	Thet, 2024 [25]
Late	Open	NR	NR
	Endovascular	1.9% (95% CI: 1.2–25%)	Cheng-Hao, 2023 [21]
	Not defined	1.3–12%	Sef, 2023 [23]

**Table 5:** Quality of evidence according to GRADE.

Q	Question	Study Design	Risk of Bias	Inconsistency	Indirectness	Publication Bias	Magnitude of Effect	Dose Response	Testing Moderators	Overall Quality	Grade
Q1	Incidence and risk factors	4	−1	−1	0	0	0	0	1	3	Moderate
Q2	Pathogenesis	2	−1	0	−1	0	0	0	0	0	Very low
Q3	Early detection	4	−1	−1	0	0	0	0	0	2	Low
Q4	Prevention	4	−1	−1	0	0	0	0	1	3	Moderate
Q5	Complications associated with CSFD	4	−1	0	0	0	0	0	0	3	Moderate
Q6	Treatment	2	−1	−1	0	0	0	0	0	0	Very low
Q7	Prognosis	2	−1	−1	0	0	0	0	0	0	Very low

GRADE, Grading of Recommendations Assessment, Development and Evaluation.

**Table 6:** Summary of the evidence.

Q	Question	Findings	Grade	Areas of Future Research
Q1	Incidence and risk factors	Open: 10%; endovascular up to 3.5%; permanent: 3.5%	Moderate	High-quality studies are needed
Q2	Pathogenesis	Disruption of the spinal cord’s blood supply at some point, leading to hypoxia, metabolic arrest, and ischemia	Very low	Pathogenetic mechanisms at the cellular and molecular level
Q3	Early detection	Neuromonitoring could be an option	Low	Optimization of neuromonitoring intraoperative, and maybe early postoperatively
Q4	Prevention	CSFD seems to work in open aortic repair, LSA F/B EVAR	Moderate	High-quality studies are needed
Q5	Complications associated with CSFD	Morbidity between 10 and 23%, non-negligible mortality	Moderate	Identify high-risk patients
Q6	Treatment	Selective CSFD, optimize perfusion and hemodynamic parameters	Very low	Study the role of several drugs, including steroids, calcium channel blockers, fasudil, and milrinone
Q7	Prognosis	Mortality of up to 75% in the first year	Very low	Studies focusing on the morbidity and mortality associated with SCI after aortic repair

## Data Availability

No new data were created or analyzed in this study.

## Abbreviations

The following abbreviations are used in this manuscript:

BMT	Best medical treatment
CSF	Cerebrospinal fluid
CSFD	Cerebrospinal fluid drain
CT	Computerized tomography
CNIHE	Canadian National Institute of Health Economics
DTA	Descending thoracic aortic
F/B-EVAR	Fenestrated or branched endovascular repair
GRADE	Grading of Recommendations Assessment
LSA	Left subclavian artery
MA	Meta-analysis
MEPs	Motor evoked potentials
MICACE	Minimally invasive segmental spinal artery coil embolization
MRI	Magnetic resonance imaging
NIRS	Near-infrared spectroscopy
NOS	Newcastle Ottawa scale
OS	Open surgery
SCI	Spinal cord ischemia
SR	Systematic review
SSEPs	Somatosensory evoked potentials
QUADAS-2	Quality Assessment of Diagnostic Accuracy Studies—2
TAAAs	Thoracoabdominal aortic aneurysms
TASP	Temporary aneurysm sac perfusion
TBAD	Type B aortic dissection
TCD	Transcranial Doppler
TEVAR	Thoracic endovascular aneurysm repair
US	Ultrasound
W/O	Without
